# Undiagnosed hypertension in Sudan: results of the cross-sectional national STEPS survey in 2016

**DOI:** 10.11604/pamj.2022.42.205.35478

**Published:** 2022-07-14

**Authors:** Supa Pengpid, Karl Peltzer

**Affiliations:** 1 Department of Health Education and Behavioral Sciences, Faculty of Public Health, Mahidol University, Bangkok, Thailand,; 2 Department of Public Health, Sefako Makgatho Health Sciences University, Pretoria, South Africa,; 3 Department of Psychology, College of Medical and Health Science, Asia University, Taichung, Taiwan,; 4 Department of Psychology, University of the Free State, Bloemfontein, South Africa

**Keywords:** Undiagnosed hypertension, adults, Sudan

## Abstract

**Introduction:**

the rate of hypertension has been increasing in Africa. The study aimed to estimate the prevalence and associated factors of undiagnosed hypertension (HTN) among adults in Sudan.

**Methods:**

cross-sectional data were analyzed from 7,226 persons (18-69 years, median 37 years, interquartile range 27-49 years, 4557 were females) who participated in the 2016 Sudan STEPS survey, had complete blood pressure measurement and non-pregnant, and responded to a questionnaire, physical measures, and biomedical tests. Logistic regression was used to determine the predictors of undiagnosed HTN.

**Results:**

the prevalence of undiagnosed HTN was 26.2% (n=2057) (79.2% of total HTN), diagnosed HTN 6.9% (n=690) and total HTN 33.1% (n=2747). In multivariable analysis older age (50-69 years) (adjusted risk ratio-aRR: 2.49, 95% CI: 2.02-3.09; p<0.001), obesity (aRR: 2.51, 95% CI: 1.97-3.21; p<0.001), diabetes (aRR: 1.59, 95% CI: 1.17-2.16; p=0.002) and elevated total cholesterol (aRR: 1.48, 95% CI: 1.19-1.84; p<0.001) were positively associated and health care advice (aRR: 0.79, 95% CI: 0.64-0.98; p=0.036) was negatively associated with undiagnosed HTN versus no HTN. Male sex (adjusted odds ratio-aOR: 2.22, 95% CI: 1.63-3.01; p<0.001) was positively associated, and older age (50-69 years) (aOR: 0.31, 95% CI: 0.21-0.46; p<0.001), married (aOR: 0.45, 95% CI: 0.33-0.62; p<0.001), urban residence (aOR: 0.70, 95% CI: 0.51-0.96; p=0.022), health care advice (aOR: 0.32, 95% CI: 0.22-0.45; p<0.001), ever cholesterol measured (aOR: 0.43, 95% CI: 0.27-0.67; p<0.001), overweight (aOR: 0.63, 95% CI: 0.47-0.85; p=0.003) and heart attack or stroke (aOR: 0.31, 95% CI: 0.17-0.55; p<0.001) were negatively associated with undiagnosed HTN versus diagnosed HTN.

**Conclusion:**

one in four adults in Sudan had undiagnosed HTN (eight in ten of total HTN) and several associated factors that can help guide interventions were identified.

## Introduction

Hypertension (or elevated blood pressure) (HTN) is a major cause of premature death worldwide. An estimated 1.28 billion adults aged 30-79 years worldwide have HTN, most (two-thirds) living in low- and middle-income countries (LMICs) [1]. Globally, 59% of women and 49% of men with HTN reported a previous diagnosis of HTN in 2019 [2]. There has been a significant increase in HTN in LMICs, yet in LMICs, only one-third of the population are aware of their HTN status [3]. Undiagnosed HTN can lead to serious morbidity, such as major cardiovascular events, cardiovascular disorders and stroke, and mortality [4,5].

In sub-Saharan Africa, based on 33 surveys, only 27% were aware of their HTN status before the surveys [6]. For example, in a rural community in Sudan, the prevalence of undiagnosed HTN was 38.2% [7], in South Africa 49% of the total HTN [8] and in Hawela Tula Sub-City, Hawassa, Southern Ethiopia, the prevalence of undiagnosed HTN was 12.3% [9]. In a small community survey among the Nubas in North Sudan, the overall prevalence of undiagnosed HTN (excluding previously diagnosed with HTN) was 49.4% [10]. There is lack of national data on the prevalence and its associated factors of undiagnosed HTN in the general adult population in Sudan.

Undiagnosed HTN can be conceptualized in problems using health services [11], using Andersen´s behavioural model of health service utilization, including predisposing factors (demographic characteristics), enabling factors (objective conditions), and need factors (perceived need for health services) [11]. Predisposing factors associated with undiagnosed HTN include younger age [5,12], older age [7,13], male sex [5,9,13], not married [13], and ethnicity [13].

Enabling/disabling factors associated with undiagnosed HTN include socioeconomic status (lower economic status [5,13], higher economic status [14], lower education [7,13,14], higher education [15]), rural residence [13], geographical region [14], no health care visit in the past month [16], and no health insurance [17]. Furthermore, health risk behaviours associated with undiagnosed HTN include physical inactivity [18], not physically inactive [13], high sedentary behaviour [19], dietary habit [19], and tobacco use [20].

The need factors associated with undiagnosed HTN include other chronic diseases, underweight [14], no obesity [5,12,16,17], obesity [7,9,13,14,18], no diabetes [21], low high-density lipoprotein-C (HDL-C), high triglycerides [22], and no cardiovascular diseases [21]. The study aimed to estimate the prevalence and associated factors of undiagnosed hypertension among people (18-69 years) in Sudan.

## Methods

**Study design and setting:** this is a cross-sectional study from the national STEPS household survey in Sudan in 2016 [23].

**Study population:** a multi-stage stratified sampling process was carried out to randomly select participants from the target population (18-69 years) in Sudan from February to December in 2016 [23]. The inclusion criteria at the household level were persons from both sexes, aged 18 to 69 years, regardless of health status and living in the selected household for 6 or more weeks [23].

**Data collection:** following the STEPS survey procedures, socio-demographic and behavioural information was collected in step 1. Physical measurements such as height, weight, and blood pressure were collected in step 2. Biochemical measurements were collected to assess blood glucose and cholesterol levels in step 3 [24]. Blood glucose and cholesterol were measured using cardio-check examination equipment (cardio check P.A. In vitro diagnostic medical devices for use with PTS panels test strips; Manufacturer: Polymer Technology Systems, INC, Indianapolis, IN USA CE 0197) [25]. The response rate for step 1 and 2 was 95%, and for step 3 was 88% [25].

**Outcome variable:** undiagnosed HTN was defined as systolic blood pressure (BP) ≥140mmHg and/or diastolic BP ≥90mmHg among people who responded “no” the question “have you ever been told by a doctor or other health worker that you have raised blood pressure or hypertension?” [25,26]. Prior to taking blood pressure measurements, participants were asked to sit quietly and rest for 15 min with legs uncrossed. Three readings of systolic and diastolic blood pressure were obtained, with participants resting for three minutes between each reading. Of the three blood pressure measurements using the Omron BP apparatus automatic blood pressure monitor; the last two readings following recommendations by World Health Organization (WHO) were averaged [25]. Predisposing factors included age, sex, and marital status [25].

Enabling or disabling factors included ever measured cholesterol, education, health care advice, current tobacco use, and physical activity. Health care advice was assessed with the question, “during the past three years, has a doctor or other health worker advised you to maintain a healthy body weight or lose weight?” (yes/no) [25]. Self-reported physical activity was assessed with the Global Physical Activity Questionnaire (GPAQ) and categorized by the median metabolic equivalent (METs) of performed activities as low, moderate, and high [27]. Need factors included Body Mass Index (BMI), diabetes, and elevated total cholesterol. BMI was classified as underweight (<18.5 kg/m^2^), normal weight (18.5-24.9 kg/m^2^), overweight (25.0-29.9 kg/m^2^), and obesity (≥30.0 kg/m^2^) [24]. Diabetes: fasting plasma glucose levels ≥7.0 mmol/L (≥126 mg/dl); or using insulin or oral hypoglycaemic drugs [24]. Elevated total cholesterol was classified [28] as: being on antilipidemic medication or having elevated total cholesterol (TC): ≥5.17 mmol/l (200 mg/dl). History of heart attack or stroke included self-reported “Have you ever had a heart attack or chest pain from heart disease (angina) or a stroke (cerebrovascular accident or incident)? (Yes, No)”.

**Ethical considerations:** ethics approval was provided by the national ethical committee at Federal Ministry of Health, Sudan. Verbal informed consent was obtained from all participants.

**Statistical analysis:** all statistical analyses were conducted with STATA software version 14.0 (Stata Corporation, College Station, TX, USA). Descriptive statistics are used to describe the sample. Multinomial logistic regression was used to estimate factors associated with undiagnosed HTN and diagnosed HTN (with not having HTN as reference category). Logistic regressions were used to assess the associations with undiagnosed HTN versus diagnosed HTN. Covariates in the logistic regression models included predisposing factors (age, gender, and marital status), enabling, or disabling factors (residence status, health care advice, cholesterol screening, education, current tobacco use, and physical activity) and need factors (BMI, diabetes, heart attack or stroke, and elevated total cholesterol). Variables significant (at p<0.1) in univariable analyses were subsequently included in the multivariable models. To account for the multi-stage sample design, Taylor linearization methods were utilized. P-values <0.05 were considered significant, and missing values (<4%) were discarded.

## Results

**Sample characteristics:** the sample with complete blood pressure measurement, excluding pregnant women, included 7,226 persons (18-69 years, median 37 years, IQR: 27-49 years, 4557 were females), in 2016. The prevalence of undiagnosed HTN was 26.2% (n=2057) (79.2% of total HTN), diagnosed HTN 6.9% (n=690) and total HTN 33.1% (n=2747). Further sociodemographic and health characteristics of the sample by HTN status are described in Table 1.

**Table 1:** sample characteristics, Sudan, 2016

Variable	Sample	No hypertension	Undiagnosed hypertension	Diagnosed hypertension
N	7226	4479	2057	690
	**N (%)**	**%**	**%**	**%**
All		66.9	26.2	6.9
**Predisposing factors**				
**Age (years)**				
18-34	3083 (56.5)	77.5	20.2	2.3
35-49	2370 (26.9)	58.7	32.2	9.1
50-69	1773 (16.5)	44.3	36.9	18.8
**Gender**				
Female	4557 (63.1)	64.7	25.5	9.8
Male	2669 (36.9)	68.7	26.7	4.6
**Marital status**				
Not married	1800 (36.4)	73.3	23.1	3.5
Married	5414 (63.6)	63.3	27.9	8.8
**Enabling/disabling factors**				
**Residence**				
Rural	4768 (62.4)	69.1	26.2	4.7
Urban	2458 (37.6)	63.3	26.2	10.4
**Health care advice (past 3 years)**				
No	6269 (87.1)	68.2	26.7	5.1
Yes	957 (12.9)	58.3	22.6	19.1
**Ever cholesterol screening**				
No	6792 (94.8)	67.9	26.3	5.8
Yes	434 (5.2)	49.4	24.9	25.7
**Education**				
None	3051 (33.6)	66.3	26.9	6.8
Primary or less	2299 (33.9)	67.9	27.0	5.1
> Primary	1864 (32.5)	66.5	24.7	8.8
Current tobacco use	811 (16.1)	67.7	26.2	6.1
**Physical activity**				
Low	1733 (21.4)	62.3	28.4	9.4
Moderate	1853 (23.3)	66.2	25.5	8.3
High	3543 (55.3)	69.0	25.8	5.2
**Need factors**				
**Body mass index**				
Normal	3631 (53.7)	71.7	24.2	4.1
Underweight	1120 (18.1)	81.1	17.0	1.9
Overweight	1535 (17.9)	52.6	34.4	13.0
Obesity	920 (10.4)	42.5	28.4	19.2
Diabetes	499 (6.0)	29.6	35.9	24.5
Heart attack or stroke	112 (1.2)	46.6	24.1	29.2
Elevated total cholesterol	933 (11.0)	48.6	35.6	15.9

**Associations with undiagnosed hypertension versus no hypertension:** in univariable analysis, older age, married, ever screened for cholesterol, underweight, overweight, obesity, diabetes and elevated total cholesterol were positively associated, and high physical activity and health care advice were negatively associated with undiagnosed versus no HTN. In multivariable analysis older age (50-69 years) (adjusted risk ratio-aRR: 2.49, 95% CI: 2.02-3.09; p<0.001), obesity (aRR: 2.51, 95% CI: 1.97-3.21; p<0.001), diabetes (aRR: 1.59, 95% CI: 1.17-2.16; p=0.002) and elevated total cholesterol (aRR: 1.48, 95% CI: 1.19-1.84; p<0.001) were positively associated and health care advice (aRR: 0.79, 95% CI: 0.64-0.98; p=0.036) was negatively associated with undiagnosed HTN versus no HTN (Table 2).

**Table 2:** associations with undiagnosed hypertension versus no hypertension

Variable	Unadjusted RR (95% CI)	P-value	Adjusted RR (95% CI)	P-value
**Predisposing factors**				
Age (years)				
18-34	1 (reference)		1 (reference)	
35-49	2.11 (1.79-2.49)	<0.001	1.81 (1.52-2.15)	<0.001
50-69	3.20 (2.66-3.86)	<0.001	2.49 (2.02-3.09)	<0.001
**Gender**				
Female	1 (reference)		1 (reference)	
Male	0.99 (0.85-1.14)	0.857	1.10 (0.94-1.29)	0.252
**Marital status**				
Not married	1 (reference)		1 (reference)	
Married	1.40 (1.17-1.67)	<0.001	0.88 (0.72-1.07)	0.171
**Enabling/disabling factors**				
**Residence**				
Rural	1 (reference)		1 (reference)	
Urban	1.09 (0.92-1.30)	0.320	0.94 (0.76-1.15)	0.679
**Health care advice (past 3 years)**				
No	1 (reference)		1 (reference)	
Yes	0.69 (0.54-0.91)	0.012	0.79 (0.64-0.98)	0.036
**Ever cholesterol screening**				
No	1 (reference)		1 (reference)	
Yes	1.30 (0.92-1.84)	<0.001	0.98 (0.68-1.41)	0.927
Education				
None	1 (reference)		1 (reference)	
Primary or less	0.98 (0.83-1.17)	0.840	0.97 (0.80-1.17)	0.766
>primary	0.92 (0.75-1.12)	0.386	0.93 (0.73-1.18)	0.539
Current tobacco use	0.99 (0.79-1.23)	0.921	---	
**Physical activity**				
Low	1 (reference)		1 (reference)	
Moderate	0.84 (0.68-1.04)	0.116	1.04 (0.81-1.33)	0.775
High	0.82 (0.69-0.97)	0.021	1.08 (0.88-1.32)	0.466
**Need factors**				
**Body mass index**				
Normal	1 (reference)		1 (reference)	
Underweight	0.62 (0.50-0.77)	<0.001	0.65 (0.52-0.83)	<0.001
Overweight	1.93 (1.61-2.32)	<0.001	1.66 (1.35-2.05)	<0.001
Obesity	2.68 (2.11-3.40)	<0.001	2.51 (1.97-3.21)	<0.001
Diabetes	2.44 (1.84-3.25)	<0.001	1.59 (1.17-2.16)	0.002
Heart attack or stroke	1.33 (0.65-2.71)	0.436	1.06 (0.47-2.35)	0.897
Elevated total cholesterol	2.02 (1.65-2.48)	<0.001	1.48 (1.19-1.84)	<0.001

RR: relative risk ratio; CI: confidence intervals

**Associations with diagnosed hypertension versus no hypertension:** in univariable analysis, older age, being married, urban residence, health care advice, ever screened for cholesterol, overweight, obesity, diabetes, heart attack or stroke and elevated total cholesterol were positively associated, and male sex, primary or less education, high physical activity and underweight were negatively associated with diagnosed HTN versus no HTN. In multivariable analysis, older age (50-69 years) (aRR: 8.19, 95% CI: 5.59-12.01; p<0.001), married (aRR: 1.71, 95% CI: 1.29-2.26; p<0.001), urban residence (aRR: 1.38, 95% CI: 1.01-1.90; p=0.048), health care advice (aRR: 2.61, 95% CI: 1.92-3.56; p<0.001), ever measured cholesterol (aRR: 2.06, 95% CI: 1.36-3.12; p=0.002), obesity (aRR: 3.22, 95% CI: 2.28-4.53; p<0.001), diabetes (aRR: 2.39, 95% CI: 1.56-3.66; p<0.001), heart attack or stroke (aRR: 2.56, 95% CI: 1.18-5.54; p=0.014), and elevated total cholesterol (aRR: 1.61, 95% CI: 1.19-2.19; p=0.003) were positively associated with diagnosed HTN versus no HTN. Male sex (aRR: 0.57, 95% CI: 0.44-0.74; p<0.001), and underweight (aRR: 0.55, 95% CI: 0.33-0.92; p=0.019) were negatively associated with diagnosed HTN versus no HTN (Table 3).

**Table 3:** associations with diagnosed hypertension versus no hypertension

Variable	Unadjusted RR (95% CI)		Adjusted RR (95% CI)	P-value
**Predisposing factors**				
**Age (years)**				
18-34	1 (reference)		1 (reference)	
35-49	5.16 (3.61-7.38)	<0.001	3.05 (2.05-4.53)	<0.001
50-69	14.10 (9.92-20.02)	<0.001	8.19 (5.59-12.01)	<0.001
**Gender**				
Female	1 (reference)		1 (reference)	
Male	0.44 (0.35-0.56)	<0.001	0.57 (0.44-0.74)	<0.001
**Marital status**				
Not married	1 (reference)		1 (reference)	
Married	2.87 (2.21-3.73)	<0.001	1.71 (1.29-2.26)	<0.001
**Enabling/disabling factors**				
**Residence**				
Rural	1 (reference)		1 (reference)	
Urban	2.41 (1.79-3.24)	<0.001	1.38 (1.01-1.90)	0.048
**Health care advice (past 3 years)**				
No	1 (reference)		1 (reference)	
Yes	4.42 (3.29-5.95)	<0.001	2.61 (1.92-3.56)	<0.001
**Ever cholesterol screening**				
No	1 (reference)		1 (reference)	
Yes	6.03 (4.23-8.61)	<0.001	2.06 (1.36-3.12)	0.002
**Education**				
None	1 (reference)		1 (reference)	
Primary or less	0.73 (0.55-0.97)	0.032	0.83 (0.61-1.12)	0.233
>primary	1.29 (0.94-1.76)	0.115	1.19 (0.84-1.69)	0.325
Current tobacco use	0.86 (0.62-1.19)	0.325	---	
**Physical activity**				
Low	1 (reference)		1 (reference)	
Moderate	0.84 (0.65-1.08)	0.166	1.11 (0.82-1.50)	0.547
High	0.51 (0.39-0.66)	<0.001	1.00 (0.74-1.36)	0.964
**Need factors**				
**Body mass index**				
Normal	1 (reference)		1 (reference)	
Underweight	0.42 (0.26-0.67)	<0.001	0.55 (0.33-0.92)	0.019
Overweight	4.32 (3.37-5.52)	<0.001	2.64 (1.97-3.55)	<0.001
Obesity	7.90 (5.81-10.74)	<0.001	3.22 (2.28-4.53)	<0.001
Diabetes	7.43 (5.15-10.72)	<0.001	2.39 (1.56-3.66)	<0.001
Heart attack or stroke	6.38 (3.59-11.32)	<0.001	2.56 (1.18-5.54)	0.014
Elevated total cholesterol	3.95 (3.01-5.16)	<0.001	1.61 (1.19-2.19)	0.003

RR: relative risk ratio; CI: confidence intervals

**Associations with undiagnosed hypertension versus diagnosed hypertension:** in univariable analysis, male sex, primary or less education, and high physical activity were positively associated, and older age, married, urban residence, health care advice, ever screened for cholesterol, more than primary education, overweight, obesity, diabetes, history of heart attack or stroke, and elevated total cholesterol were negatively associated with undiagnosed HTN versus diagnosed HTN. In multivariable analysis, male sex (adjusted odds ratio-aOR: 2.22, 95% CI: 1.63-3.01; p<0.001) was positively associated, and older age (50-69 years) (aOR: 0.31, 95% CI: 0.21-0.46; p<0.001), married (aOR: 0.45, 95% CI: 0.33-0.62; p<0.001), urban residence (aOR: 0.70, 95% CI: 0.51-0.96; p=0.022), health care advice (aOR: 0.32, 95% CI: 0.22-0.45; p<0.001), ever cholesterol measured (aOR: 0.43, 95% CI: 0.27-0.67; p<0.001), overweight (aOR: 0.63, 95% CI: 0.47-0.85; p=0.003) and heart attack or stroke (aOR: 0.31, 95% CI: 0.17-0.55; p<0.001) were negatively associated with undiagnosed HTN versus diagnosed HTN (Table 4).

**Table 4:** associations with undiagnosed hypertension versus diagnosed hypertension

Variable	Unadjusted ORs (95% CI)	P-value	Adjusted ORs (95% CI)	P-value
**Predisposing factors**				
**Age (years)**				
18-34	1 (reference)		1 (reference)	
35-49	0.41 (0.28-0.59)	<0.001	0.67 (0.44-1.03)	0.056
50-69	0.23 (0.16-0.33)	<0.001	0.31 (0.21-0.46)	<0.001
**Gender**				
Female	1 (reference)		1 (reference)	
Male	2.22 (1.74-2.82)	<0.001	2.22 (1.63-3.01)	<0.001
**Marital status**				
Not married	1 (reference)		1 (reference)	
Married	0.49 (0.36-0.66)	<0.001	0.45 (0.33-0.62)	<0.001
**Enabling/disabling factors**				
**Residence**				
Rural	1 (reference)		1 (reference)	
Urban	0.45 (0.34-0.61)	<0.001	0.70 (0.51-0.96)	0.022
**Health care advice (past 3 years)**				
No	1 (reference)		1 (reference)	
Yes	0.22 (0.16-0.31)	<0.001	0.32 (0.22-0.45)	<0.001
**Ever cholesterol screening**				
No	1 (reference)		1 (reference)	
Yes	0.22 (0.15-0.31)	<0.001	0.43 (0.27-0.67)	<0.001
**Education**				
None	1 (reference)		1 (reference)	
Primary or less	1.34 (1.01-1.78)	0.043	1.12 (0.81-1.54)	0.486
>primary	0.71 (0.52-0.97)	0.032	0.76 (0.53-1.07)	0.101
Current tobacco use	1.15 (0.82-1.62)	0.412	---	
**Physical activity**				
Low	1 (reference)		1 (reference)	
Moderate	1.01 (0.76-1.34)	0.939	0.93 (0.66-1.31)	0.657
High	1.62 (1.22-2.15)	<0.001	1.08 (0.78-1.48)	0.657
**Need factors**				
**Body mass index**				
Normal	1 (reference)		1 (reference)	
Underweight	1.48 (0.90-2.42)	0.120	1.17 (0.69-1.99)	0.578
Overweight	0.45 (0.34-0.59)	<0.001	0.63 (0.47-0.85)	0.003
Obesity	0.34 (0.25-0.45)	<0.001	0.83 (0.57-1.20)	0.301
Diabetes	0.33 (0.24-0.46)	<0.001	0.67 (0.45-1.02)	0.058
Heart attack or stroke	0.21 (0.10-0.42)	<0.001	0.31 (0.17-0.55)	<0.001
Elevated total cholesterol	0.51 (0.39-0.67)	<0.001	0.92 (0.67-1.27)	0.603

OR: odds ratio; CI: confidence intervals

## Discussion

The study aimed to estimate the prevalence and associated factors of undiagnosed hypertension among people (18-69 years) in Sudan. One in four adults in Sudan had undiagnosed HTN (eight in ten of total HTN). Predisposing factors (older age), enabling factors (no health care advice) and need factors (obesity, diabetes, and elevated total cholesterol) were associated with undiagnosed HTN versus no HTN, and predisposing factors (younger age, not married, and male sex), enabling factors (rural residence, no health care advice, never cholesterol measured), and need factors (not overweight and no history of heart attack or stroke) were identified as associated with undiagnosed HTN versus diagnosed HTN.

The prevalence of undiagnosed HTN in Sudan (26.2%, 79.2% of total HTN), was higher than in a review of 33 surveys in sub-Saharan Africa (73% of HTN) [6], higher than in a rural community in Sudan (38.2% of HTN) [7], in South Africa (49% of HTN) [8] and higher than in Southern Ethiopia (12.3%) [9], but lower than in a small community survey among the Nubas in North Sudan (49.4% excluding previously diagnosed with HTN) [10]. Possible reasons for the high prevalence of undiagnosed HTN in Sudan may include lack of awareness of routine blood pressure screening [7,10]. People with undiagnosed HTN versus diagnosed HTN showed less HTN-related comorbidities, such as older age and obesity, and in unadjusted analysis diabetes and elevated total cholesterol, than those with diagnosed HTN. This may be explained by the fact that people with undiagnosed HTN are generally younger and healthier than those with diagnosed HTN, mainly at an earlier stage of HTN, people with chronic diseases, such as obesity and heart attack or stroke, are more likely to use health care services, increasing the chances of diagnosis of HTN [29].

Consistent with some previous research [5,7,9,10,12,13,17,18], some predisposing factors (male sex, older age, and not married) were associated with undiagnosed HTN versus no HTN or diagnosed HTN in this study. In terms of sex, men are less likely to visit health facilities than women, which can increase their likelihood of having undiagnosed HTN [29]. The finding that persons who were married were less likely to have undiagnosed HTN may be explained by a higher likelihood of couples utilization health care services, including preventive screening, than singles [29]. Screening programmes to identify HTN should be targeted at men, older adults and singles.

According to some previous studies [13,16,20], enabling/disabling factors associated with undiagnosed HTN included not having screened for cholesterol, rural residence, and no health care advice. People who have tested for cholesterol and/or received health care advice are more likely to use health services and, consequently, may reduce the odds of undiagnosed HTN [16]. Rural residents in Sudan may have poorer access to health care services than urban residents, hindering them from screening of blood pressure [30]. Contrary to several previous studies [5,7,13-15], we did not find an association between socioeconomic status (education) with undiagnosed HTN.

Consistent with some research [7,9,13,14,18,22] the need factors associated with undiagnosed HTN included other chronic diseases, such as obesity, diabetes, and elevated total cholesterol. It is well documented how excess body weight and elevated cholesterol levels contribute to HTN [22]. Educational programmes are indicated to create awareness of maintaining healthy body weight and cholesterol levels [13]. Some previous research [21] showed an association between no diabetes with undiagnosed HTN, which we found in unadjusted analysis with undiagnosed HTN versus diagnosed HTN. Policy implications are that increased public awareness campaigns, and screening of HTN are needed to reduce undiagnosed HTN in Sudan [10,31]. Health systems through primary healthcare need to be strengthened and universal health coverage to prevent and manage NCDs, including hypertension [32].

The study strengths include the use of a nationally representative sample and standardized STEPS methodology and measures. Some variables were evaluated by self-report, which may have biased responses, and the cross-sectional design precludes causative conclusions between the evaluated variables. The sample only included persons 18-69 years and those who non-institutionalized, while the inclusion of 70 years and older and institutionalized persons would have given different estimates. Furthermore, certain variables, such as knowledge of the symptoms of HTN and a family history of HTN, were not evaluated and should be included in future research.

## Conclusion

One in four adults in Sudan had undiagnosed HTN (eight in ten of total HTN). Predisposing factors (older age), enabling factors (no health care advice) and need factors (obesity, diabetes and elevated total cholesterol) were associated with undiagnosed HTN versus no HTN, and predisposing factors (younger age, not married, and male sex), enabling factors (rural residence, no health care advice, never cholesterol measured), and need factors (not overweight and no history of heart attack or stroke) were identified as associated with undiagnosed HTN versus diagnosed HTN, which can be targeted in interventions.

### What is known about this topic

- *Hypertension (or elevated blood pressure) (HTN) is a major cause of premature death;*
- *undiagnosed HTN can lead to serious morbidity*.

### What this study adds

- *One in four adults in Sudan had undiagnosed HTN (eight in ten of total HTN);*
- *predisposing factors (younger age, not married, and male sex), enabling factors (rural residence, no health care advice, never cholesterol measured), and need factors (not overweight and no history of heart attack or stroke) were associated with undiagnosed HTN versus diagnosed HTN*.
